# Improvement of indirect immunofluorescence technique to detect antineutrophil cytoplasmic antibodies and its impact on test positivity rate

**DOI:** 10.1590/1414-431X2023e12636

**Published:** 2023-07-21

**Authors:** E.B. Zarur, S.H. Rodrigues, O. Ambrogini, M.L.G. Ferraz, G. Mastroianni-Kirsztajn, L.E.C. Andrade, A.W.S. de Souza

**Affiliations:** 1 Divisão de Reumatologia, Departamento de Medicina, Escola Paulista de Medicina, Universidade Federal de São Paulo, São Paulo, SP, Brasil; 2 Divisão de Gastroenterologia, Departamento de Medicina, Escola Paulista de Medicina, Universidade Federal de São Paulo, São Paulo, SP, Brasil; 3 Divisão de Nefrologia, Departamento de Medicina, Escola Paulista de Medicina, Universidade Federal de São Paulo, São Paulo, SP, Brasil

**Keywords:** Antineutrophil cytoplasmic antibodies, ANCA-associated vasculitis, Autoimmune hepatitis, Ulcerative colitis, PR3-ANCA, MPO-ANCA

## Abstract

The indirect immunofluorescence (IIF) technique for antineutrophil cytoplasmic antibodies (ANCA) detection is subject to substantial differences across laboratories. This study aimed to assess the impact of improvements in the IIF-ANCA technique on the positivity rate of ANCA tests. A cross-sectional study was performed with serum samples from patients with ANCA-associated vasculitis (AAV), autoimmune hepatitis (AIH), and ulcerative colitis (UC). A paired analysis was performed for IIF-ANCA results using the traditional method and a modified protocol after a series of specific adjustments in the technique based on the protocol of IIF-ANCA test performed at a nation-wide private laboratory in Brazil. ANCA specificity was assessed by ELISA for anti-proteinase 3 (PR3) and anti-myeloperoxidase (MPO) antibodies. Sixty-one patients were evaluated. The positivity rate of IIF-ANCA tests at disease presentation performed at the University reference laboratory was 32.3% in AAV, AIH, and UC patients, whereas the positivity rates of IIF-ANCA and ELISA tests in other laboratories were 75.0 and 72.7%, respectively. After modifications in the IIF-ANCA technique, there was a significant increase in the positivity rate (14.8 *vs* 34.3%; P=0.0002) and in median titers [1/40 (1/30-1/160) *vs* 1/80 (1/40-1/80); P=0.0003] in AAV, AIH, and UC patients. UC had the highest increment in positive results from 5.3 to 36.8%. There was poor agreement between MPO- or PR3-ANCA and both IIF-ANCA techniques. In conclusion, modifications in the IIF-ANCA protocol led to a significant improvement in its positivity rate and titers.

## Introduction

Antineutrophil cytoplasmic antibodies (ANCA) are biomarkers of a group of systemic small vessel necrotizing vasculitis, named ANCA-associated vasculitis (AAV), which encompasses granulomatosis with polyangiitis (GPA), microscopic polyangiitis (MPA), eosinophilic granulomatosis with polyangiitis (EGPA), and renal limited vasculitis (RLV), but might also be detected in other diseases such as ulcerative colitis (UC), type 1 autoimmune hepatitis (type 1 AIH), and primary sclerosing cholangitis (1-5).

Methods for ANCA detection include indirect immunofluorescence (IIF), where the fluorescence pattern and titer are described, and solid-phase immunoassays to detect ANCA specificity, i.e., anti-proteinase 3 (PR3)-ANCA or anti-myeloperoxidase (MPO)-ANCA. The IIF patterns include cytoplasmic (C-ANCA), perinuclear (P-ANCA), atypical (A-ANCA), and atypical cytoplasmic (atypical C-ANCA). PR3-ANCA are generally associated to C-ANCA and MPO-ANCA to P-ANCA (4,6,7).

For decades, the in-house IIF-ANCA technique was the only method available to detect ANCA at the Immuno-Rheumatology Laboratory at the University hospital that serves the national reference center for vasculitis in Brazil. However, the IIF-ANCA results at this reference center have displayed a positivity rate considerably lower than that observed in the literature. Faced with this problem, our team set out to review the methodological details of the test and propose a series of modifications aiming to improve the performance of the test. This study assesses the impact of these modifications in the IIF-ANCA technique on the positivity rate and titer obtained in patients with diseases known to bear positive ANCA tests. Additionally, the agreement between both IIF-ANCA techniques and a third generation ELISA assay was evaluated.

## Material and Methods

### Serum samples

A cross-sectional study was performed including sixty-one patients with ANCA-associated diseases from the Rheumatology and Gastroenterology/Hepatology outpatient clinics at the University hospital (Universidade Federal de São Paulo). Blood samples were collected from January 2018 until December 2018. Serum samples were stored in cryotubes at -20°C. ANCA testing was performed in two steps: first, sera from study participants underwent IIF-ANCA following the Immuno-Rheumatology Laboratory standard protocol and materials. Then, improvements in the IIF-ANCA technique were applied and a second IIF-ANCA test was performed using the same serum samples. ANCA specificity by MPO-ANCA and PR3-ANCA testing was also evaluated using the same samples (Supplementary Figure S1).

The following ANCA-associated diseases were included in the study: AAV, which encompasses GPA, EGPA, MPA and RLV, UC, and type I AIH. Inclusion criteria were age ≥18 years, meeting the European Medicines Agency (EMEA) criteria for AAV (8): biopsy proven pauci-immune glomerulonephritis regardless of ANCA or MPO/PR3-ANCA positivity without any systemic manifestation for RLV (8), the clinical, radiological, and histological criteria (Lennard-Jones criteria) for UC (9), and the revised diagnostic criteria for AIH of the international group of autoimmune hepatitis or antinuclear antibody (ANA) positivity and/or anti-smooth muscle positivity at titers ≥1/80 for type I AIH (10). Disease activity of AAV patients was assessed by the third version of Birmingham Vasculitis Activity Score (BVAS) (11) and information about therapy and ANCA status at diagnosis was collected from patients' medical records. Written informed consent was obtained from all participants and the study protocol was approved by the Ethics Committee of the University hospital.

### Standard IIF-ANCA technique

The slides used were prepared in-house, using peripheral blood samples from a healthy donor and the following materials: Histopaque^®^ (Merck, Germany), FITC-labeled monoclonal antibodies IgG goat anti-human Fluocon^®^ (Wama Diagnostica, Brazil), frosted slides and coverslips from Precision^®^ Glass Line (Precision Glass, Canada), and the Olympus^®^ IIF microscope available at the reference Immuno-Rheumatology Laboratory. The healthy donor was a 46-year-old White man, with no comorbidities or illicit drug use, no active or chronic infectious diseases, and no long-term therapy.

Polymorphonuclear leukocytes were separated from blood samples using Histopaque^®^, washed 3 times with 10 mL PBS with a 10-min centrifugation at 900 *g* and 22°C each time. The neutrophil pellet was aspirated and mixed with a 20 mL PBS solution with 1% human albumin. Finally, the cells were counted on a slide and a count of 5 cells/400× magnification field was considered satisfactory.

Cytocentrifugation was used to mount slides with two spots of cytocentrifuged cells. The neutrophil cell suspension (50 μL) was added to the cuvettes attached to glass slides and paper pads in the cytocentrifuge. The solution was spun at 130 *g* and 22°C for 5 min. Glass slides were extracted from the cytocentrifuge and the area around the cytocentrifuged cells was encircled with a permanent marker. Afterwards, the slides were fixed in ethanol at -20°C for 5 min or in 4% formalin for 30 min, the latter slides were used to differentiate ANA positivity from P-ANCA. An IIF-ANCA test was considered positive if there was distinctive reactivity at titer of 1/20 or greater and the patterns recognized were C-ANCA, P-ANCA, A-ANCA, and atypical C-ANCA.

### Improvement of IIF-ANCA technique

The following modifications were based on technical issues detected during critical analysis of the IIF-ANCA standard procedures performed in our laboratory, including: 1) too many lymphocytes and few neutrophils on some slides; 2) intense background on slides; and 3) blurry images through the microscope lenses. Table 1 depicts the main differences between the standard and the improved IIF-ANCA technique.

**Table 1:** Differences between standard and improved indirect immunofluorescence-antineutrophil cytoplasmic antibodies (IIF-ANCA) techniques.

Materials/items	Standard technique	Improved technique
Neutrophil separation solution	Hystopaque^®^ (Merck, Germany)	PlymorphPrep™ (Progen, Germany)
Glass slides	Frosted Slides, Precision^®^ Glass Line (Precision Glass, Canada)	Plain polished slides, Perfecta^®^ (Perfecta, Brazil)
Conjugated antibody	FITC-labeled monoclonal antibodies goat anti-human IgG Fluocon^®^ (Wama DiagnosticaBrazil)	Goat anti-human IgG light and heavy chains from Bion MBL^®^ (Aesku Group, Germany)
IIF microscope	-	Specialized review of IIF microscope components

IIF: Indirect immunofluorescence.

The protocol steps to prepare the IIF-slides remained the same, however four materials used in the standard protocol were modified. All modifications were based on a previously published protocol (12-17).

In the process of isolating polymorphonuclear cells, Histopaque^®^ was switched to PolymorphPrep™ (Progen, Germany), which specifically separates polymorphonuclear cells, while Histopaque^®^ does not distinguish mono- from polymorphonuclear cells. The conventional glass slides were replaced by the new type of plain polished slides from Perfecta^®^ (Perfecta, Brazil) and the conjugated antibodies were changed to the goat anti-human IgG light and heavy chains from Bion MBL^®^ (Aesku Group, Germany). Finally, the IIF microscope was serviced, its items were checked, lenses were cleaned, and the filter, lamps, and objectives were replaced.

### ELISA technique

MPO-ANCA and PR3-ANCA were tested using a third generation ELISA kit (Euroimmun, Germany). All procedures were done according to the manufacturer's manual and the curve was built using Microsoft Excel software with calibrator parameters. Absorbance reading was performed at 450 nm wavelength, with a reference between 620 and 650 nm. Results were converted from absorbance to relative units (RU) per mL. Values above 20 RU/mL were considered positive for both MPO-ANCA and PR3-ANCA.

### Statistical analysis

Categorical variables are reported as absolute number and percentage, while numerical variables are reported as median and interquartile range or mean and standard deviation, accordingly. Comparisons between categorical variables were made with the chi-squared test and Fisher's exact test. Continuous variables were compared between two groups with Student's *t*-test or by the Mann-Whitney U test and between three groups with one-way ANOVA or Kruskal-Wallis test. *Post hoc* analyses were done with Scheffé test or the Mann-Whitney U test. Kappa statistics was used to analyze the agreement of categorical data, and longitudinal data were assessed with McNemar's test for categorical variables and with Wilcoxon test for paired data. A P-value <0.05 was considered statistically significant, and for multiple comparisons done by the *post hoc* Mann-Whitney U test, an adjusted P-value <0.016 was used. Data were analyzed using IBM SPSS (USA) for Windows 21.0 and GraphPad Prism (USA) for Windows 5.0.

## Results

### Subjects' characteristics

Sixty-one samples of patients with diseases associated with ANCA were included in the study. Table 2 depicts demographic characteristics, diagnosis of study participants, and therapy at study. Type 1 AIH patients were significantly younger than AAV patients (P=0.024), and the percentage of females was higher in type 1 AIH patients compared to AAV patients (P=0.010). Amongst AAV patients, 18 (60.0%) had GPA, six (20.0%) had MPA, five (16.6%) had EGPA and only one (3.3%) patient had RLV. The median time elapsed since diagnosis was significantly lower in the AAV group (47.5 months) compared with type 1 AIH (108.0 months, P=0.012) and with UC patients (84.0 months, P=0.025). Only two patients were taking ANCA-inducing medications: one was on allopurinol and the other one was taking both allopurinol and hydralazine. The former patient had a negative ANCA test with the standard technique and a positive 1/40 C-ANCA with the improved IIF technique while the latter patient had a positive 1/160 C-ANCA on both in-house techniques. PR3-ANCA was also positive in both patients and neither had a positive test at diagnosis. No acute or chronic infectious diseases or illicit drug use were found in study participants.

**Table 2:** Demographic features and treatment of study participants.

Variables	AAV (n=30)	Type 1 AIH (n=12)	UC (n=19)	P
Age, years	55.7±11.5	40.8±19.3	46.3±18.2	0.013
Females, n (%)	18 (60.0)	12 (100.0)	15 (78.9)	0.024
Time since diagnosis, months	47.5 (18.5-96.0)	108.0 (72.0-132.0)	84.0 (43.0-168.0)	0.016
Therapies				
Prednisone, n (%)	18 (60.0)	5 (41.7)	2 (7.8)	0.003
Prednisone, mg/day	15.0 (5.0-30.0)	10.0 (10.0-15.0)	30.0 (20.0-40.0)	0.467
Mesalazine, n (%)	0	0	13 (68.4)	NA
Immunosuppressive agents, n (%)	13 (43.3)	10 (83.3)	3 (15.7)	0.001
Azathioprine, n (%)	4 (13.3)	9 (75.0)	3 (15.7)	NA
Methotrexate, n (%)	3 (10.0)	0	0	NA
Oral cyclophosphamide, n (%)	3 (10.0)	0	0	NA
*iv* Cyclophosphamide, n (%)	2 (6.6)	0	0	NA
Leflunomide, n (%)	1 (3.3)	0	0	NA
Mycophenolate sodium, n (%)	0	1 (8,3)	0	NA
Biologic agents, n (%)	5 (16.7)	1 (8.3)	4 (21.1)	0.647
Rituximab, n (%)	5 (16.7)	0	0	NA
TNFα antagonists, n (%)	0	1 (8.3)	4 (21.1)	NA

AAV: ANCA-associated vasculitis; AIH: autoimmune hepatitis; UC: ulcerative colitis; *iv*: intravenous; n: number of patients; NA: not applicable; TNF: tumor necrosis factor. Comparisons between categorical variables were made with the chi-squared test and Fisher's exact test. Continuous variables were compared between three groups with one-way ANOVA or Kruskal-Wallis test.

Among AAV patients, the mean BVAS v3 score at diagnosis was 14.6±6.1, and at study inclusion only 8 patients presented active vasculitis with a mean BVAS v3 score of 6.3±3.6.

Prednisone was used by 41% of all patients, more frequently in AAV patients than in UC (P=0.0008). However, the median prednisone daily dose was similar among all groups. Immunosuppressants were used by approximately 43% of all patients, especially by type 1 AIH patients, where the frequency of use of these drugs was higher than in AAV (P=0.037) and UC patients (P=0.0005). Only 16% of the patients were using biologic agents, represented by rituximab in AAV patients and by tumor necrosis factor alpha (TNFα) antagonists for UC therapy. The only patient in the AIH group on TNFα antagonist therapy also had Crohn's disease.

### ANCA results at diagnosis

IIF-ANCA test was performed in only half of the study participants by the Immuno-Rheumatology laboratory at diagnosis: 25 (83.3%) AAV patients, 3 (25.0%) type 1 AIH, and 3 (25.8%) UC patients. The positivity rate for IIF-ANCA tests in those patients was 32.3% and it was more frequently observed in AAV patients. Twelve patients also had IIF-ANCA test done at a private laboratory, with a positivity rate of 75.0%. The IIF pattern and positivity rate of ANCA tests performed at diagnosis in each disease are shown on Table 3.

**Table 3:** Indirect immunofluorescence-antineutrophil cytoplasmic antibodies (IIF-ANCA) features of tests performed at the Immuno-Rheumatology laboratory and in independent laboratories at diagnosis.

IIF-ANCA	GPA	MPA	EGPA	RLV	Type 1 AIH	UC
Tests performed at Immuno-Rheumatology laboratory	15	4	5	1	3	3
Positive tests, n (%)	5 (33.3)	2 (50.0)	0 (0.0)	1 (100.0)	1 (33.3)	1 (33.3)
C-ANCA, n (%)	5	0	0	0	0	1
P-ANCA, n (%)	0	1	0	1	0	0
A-ANCA, n (%)	0	1	0	0	1	0
Tests performed in outside laboratories	7	3	1	0	1	0
Positive tests, n (%)	5 (71.4)	3 (100.0)	0 (0.0)	-	1 (100.0)	-
C-ANCA, n (%)	4	2	0	-	0	-
P-ANCA, n (%)	1	1	0	-	0	-
A-ANCA, n (%)	0	0	0	-	1	-

AIH: autoimmune hepatitis; EGPA: eosinophilic granulomatosis with polyangiitis; GPA: granulomatosis with polyangiitis; MPA: microscopic polyangiitis; RVL: renal limited vasculitis; UC: ulcerative colitis; n: number of patients. The total number of tests performed at the Immuno-Rheumatology laboratory was 31 while 12 tests were performed in independent laboratories at diagnosis.

**Figure 1:**
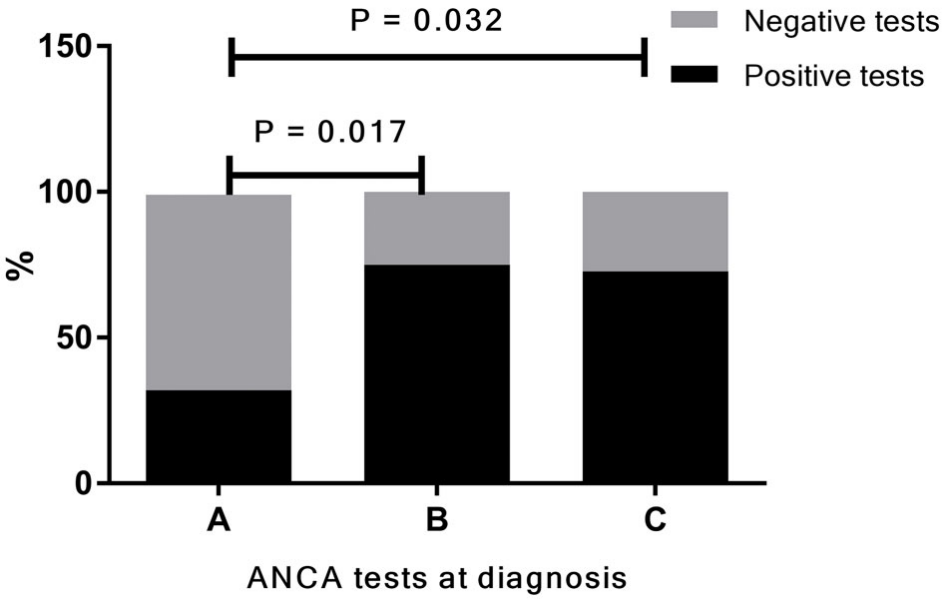
Positivity of antineutrophil cytoplasmic antibodies (ANCA) tests performed at diagnosis. This analysis includes all study participants that had ANCA tests performed at diagnosis. The positivity rate was 32.0% (10/31) for indirect immunofluorescence (IIF)-ANCA tests performed at the Immuno-Rheumatology laboratory (**A**), 75.0% (9/12) for IIF-ANCA tests performed by other laboratories (**B**), and 72.8% (8/11) for ELISA tests performed at diagnosis by other laboratories (**C**). Analyses were performed with Fisher's exact test.

Eleven patients had ANCA specificity by MPO-ANCA and PR3-ANCA tested at diagnosis, of which 10 had AAV (6 GPA, 3 MPA, and 1 EGPA) and only one had UC. The overall positivity of the ELISA tests was 72.7%. Three patients with GPA and 2 with MPA were PR3-ANCA-positive. Two patients with MPA had a positive MPO-ANCA, and one of those tested positive for both PR3 and MPO-ANCA. The only EGPA patient tested for ANCA specificity at diagnosis had a positive MPO-ANCA. Finally, the single patient with UC that tested for ANCA specificity at diagnosis also had positivity for both MPO and PR3-ANCA. The positivity rates of IIF-ANCA (75.0%) and ELISA results (72.8%) done on the non-university private laboratory were significantly higher than the positivity rate of IIF-ANCA performed at the Immuno-Rheumatology laboratory (32.0%) as shown in Figure 1 (P=0.017 and P=0.032; respectively).

### ANCA tests at study

#### ANCA tests performed prior to improvements in the IIF technique

**Figure 2:**
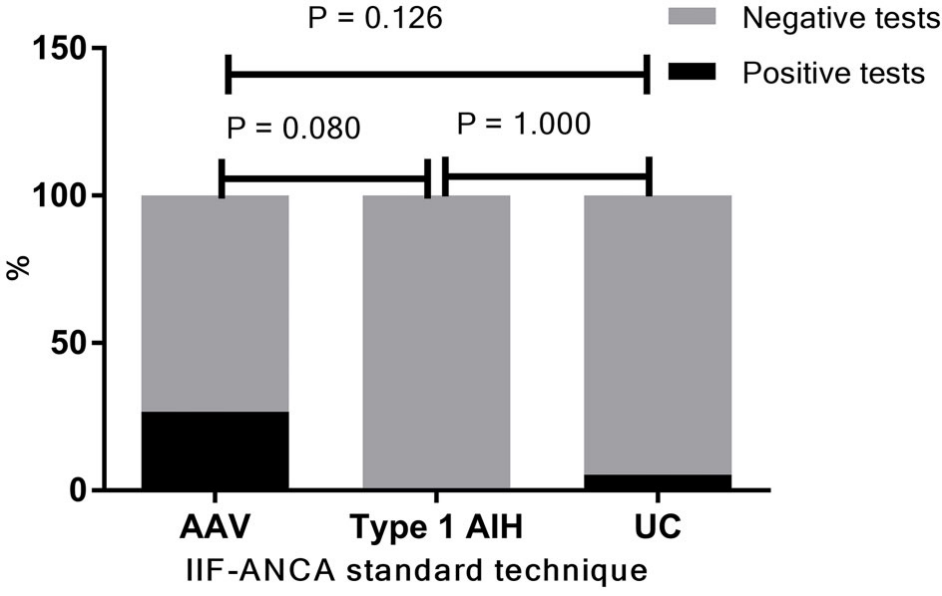
Positivity of indirect immunofluorescence-antineutrophil cytoplasmic antibodies (IIF-ANCA) tests using the standard technique. The IIF-ANCA using the standard technique was positive in 26.7% (8/18) of ANCA associated vasculitis (AAV) patients and in 5.3% (1/19) of ulcerative colitis (UC) patients, while no patient with type 1 autoimmune hepatitis (type 1 AIH) had a positive test. Analyses were performed with Fisher's exact test.

The overall positivity of IIF-ANCA performed with the standard protocol was 14.8% and the majority of positive tests occurred in samples from AAV patients (26.7%) followed by UC (5.3%), whereas no type 1 AIH patient had a positive IIF-ANCA test (Figure 2). C-ANCA was the most common pattern, followed by P-ANCA. No patient presented the A-ANCA pattern at this step (Table 4).

**Table 4:** Indirect immunofluorescence-antineutrophil cytoplasmic antibodies (IIF-ANCA) results and diseases before and after technique improvements.

Diseases	Standard technique				Improved technique			
	Positivity	C-ANCA	P-ANCA	A-ANCA	Positivity	C-ANCA	P-ANCA	A-ANCA
GPA	8/18 (44.4%)	6 (75.0%)	2 (25.0%)	0	9/18 (50.0%)	6 (66.7%)	3 (33.3%)	0
MPA	0	0	0	0	1/6 (16.7%)	1 (100.0%)	0	0
EGPA	0	0	0	0	1/5 (20.0%)	1 (100.0%)	0	0
RLV	0	0	0	0	0	0	0	0
Type 1 AIH	0	0	0	0	3/12 (25.0%)	2 (66.7%)	0	1 (33.3%)
UC	1/19 (5.3%)	0	1 (100.0%)	0	7/19 (36.8%)	4 (57.1%)	1 (14.3%)	2 (28.6%)

AIH: autoimmune hepatitis; EGPA: eosinophilic granulomatosis with polyangiitis; GPA: granulomatosis with polyangiitis; MPA: microscopic polyangiitis; RVL: renal limited vasculitis; UC: ulcerative colitis.

#### ANCA tests performed after improvements in the IIF technique

**Figure 3:**
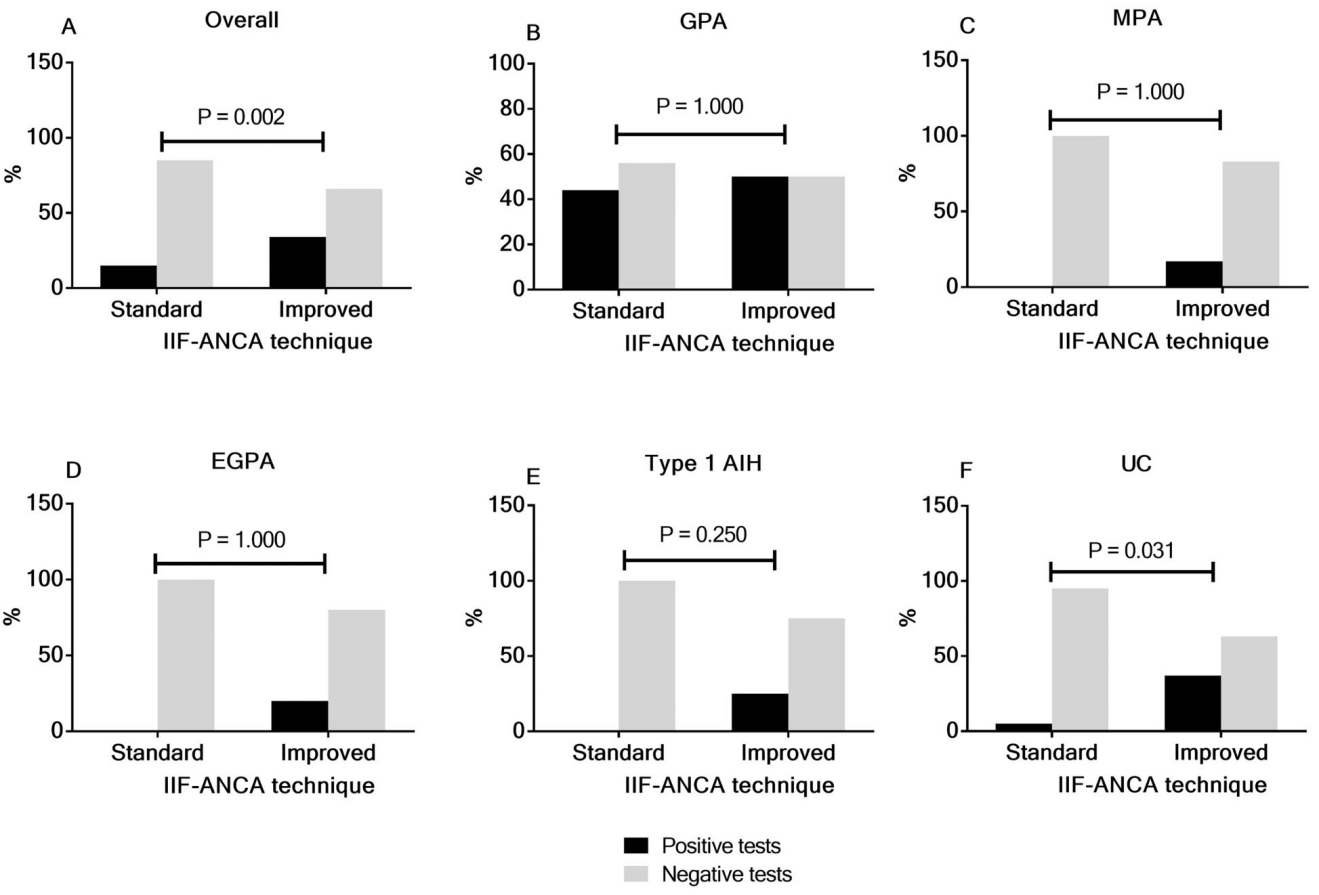
The positivity of indirect immunofluorescence-antineutrophil cytoplasmic antibodies (IIF-ANCA) tests using standard and improved techniques in each disease. The overall positivity rate improved significantly from the standard to the improved technique (**A**) [10/61(16%) to 21/61(34%)]. Although the positivity rate of IIF-ANCA tests remained stable in granulomatosis with polyangiitis (GPA) patients (**B**) [8/18(44%) to 9/18(50%)], it improved in microscopic polyangiitis (MPA) patients (**C**) [0/6 (0%) to 1/6 (17%)], in eosinophilic granulomatosis with polyangiitis (EGPA) patients (**D**) [0/5 (0%) to 1/5 (20%)], in type 1 autoimmune hepatitis (AIH) patients (**E**) [0/12 (0%) to 3/12 (25%)], and in ulcerative colitis (UC) patients (**F**) [1/19 (5%) to 7/19 (37%)]. Statistical analyses were performed with McNemar's test.

**Figure 4:**
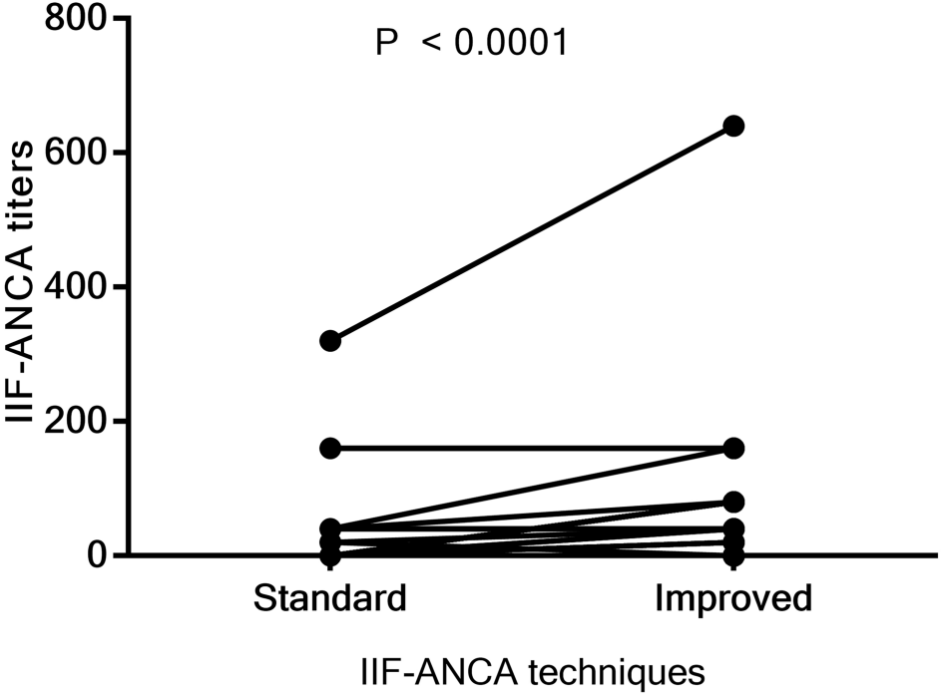
Changes in indirect immunofluorescence-antineutrophil cytoplasmic antibodies (IIF-ANCA) titers using the standard and improved IIF techniques. The median IIF-ANCA titers among positive tests increased from 1/40 (1/30-1/160) to 1/80 (1/40-1/80) using the standard and the improved IIF techniques, respectively. Data were analyzed with the Wilcoxon signed-rank test.

The overall positivity of ANCA tests increased significantly from 14.8 to 34.3% after improvements in the IIF-ANCA technique (P=0.0002). Figure 3 compares the positivity on both IIF tests in the various diseases. Positivity of ANCA tests increased from 0 to 16.7-25.0% in patients with MPA, EGPA, and type 1 AIH, but it remained stable in GPA patients. In UC patients, ANCA positivity increased from 5.3 to 36.8%. Furthermore, a significant increment in the median IIF-ANCA titer was observed after the improvements in the IIF method [1/40 (1/30-1/160) *vs* 1/80 (1/40-1/80); P=0.0003] (Figure 4). Table 4 shows the patterns and positivity rate of IIF-ANCA before and after improvements. The A-ANCA pattern was only observed in patients with UC and type 1 AIH when improvements in the method were applied.

#### Comparison of IIF-ANCA with PR3-ANCA and MPO-ANCA

The overall MPO- and PR3-ANCA positivity rate was of 16.4% in all samples. A positive PR3-ANCA result (9; 14.8%) was more frequent than MPO-ANCA (2; 3.3%). Out of ten patients presenting a positive ELISA result, six had GPA and all of them had a positive PR3-ANCA, one had MPA and tested positive for both MPO- and PR3-ANCA, one patient with type 1 AIH had a positive MPO-ANCA, and two UC patients had positive PR3-ANCA.

Despite the similar positivity rate in standard IIF-ANCA (14.8%) and in ELISA (16.4%), a poor agreement rate was observed between the two tests for the whole group of patients (kappa coefficient=0.065) and for AAV patients (kappa coefficient=0.024). This low concordance rate remained when using improved IIF-ANCA method for all patients (kappa coefficient=0.046), but a slightly better agreement was observed when only AAV patients were analyzed (kappa coefficient=0.223). Using the standard technique, most positive ELISA results were found in patients presenting negative IIF-ANCA results. After improvements in the technique, five out of 11 positive ELISA results were found in samples presenting C-ANCA pattern in IIF (Table 5).

**Table 5:** Relationship between indirect immunofluorescence (IIF) results and ELISA results ANCA detection before and after improvements in IIF techniques.

	Standard technique		Improved technique	
IIF-Patterns	PR3-ANCA+	MPO-ANCA+	PR3-ANCA+	MPO-ANCA+
Negative	7/52	2/52	5/40	1/40
C-ANCA	2/6	0	4/14	1/14
P-ANCA	0/3	0/3	0/4	0/4
A-ANCA	0/0	0/0	0/3	0/3

ANCA: antineutrophil cytoplasmic antibodies; MPO: myeloperoxidase; PR3: proteinase 3; C-ANCA: cytoplasmic ANCA; P-ANCA: perinuclear ANCA; A-ANCA: atypical ANCA.

### Impact of disease activity and therapy on ANCA results in AAV patients

Only eight AAV patients (13.1%) presented active disease at blood draw, six of them had GPA and two had EGPA. Among them, only GPA patients tested positive, with the following distribution: 16.6% for IIF-ANCA and 66.6% for PR3-ANCA.

The median daily prednisone dose was 30 mg (IQR: 2.5-60.0) in all but one AAV patient with active disease. Oral or IV cyclophosphamide or rituximab were used by five (62.5%) out of eight AAV patients with active disease (Table 6).

**Table 6:** Positivity of antineutrophil cytoplasmic antibodies (ANCA) tests, disease activity score and therapy in AAV patients presenting active disease at study.

AAV	BVAS score	IIF-ANCA standard technique	IIF-ANCA improved technique	ELISA results	Prednisone daily dose (mg)	Therapy
GPA	2	C-ANCA 1/160	C-ANCA 1/160	PR3-ANCA	2.5	-
GPA	5	Negative	Negative	PR3-ANCA	60.0	Oral cyclophosphamide
GPA	9	Negative	Negative	PR3-ANCA	30.0	Oral cyclophosphamide
GPA	6	Negative	Negative	PR3-ANCA	60.0	*iv* cyclophosphamide
EGPA	4	Negative	Negative	Negative	20.0	Rituximab
EGPA	3	Negative	Negative	Negative	2.5	Rituximab
GPA	13	Negative	Negative	Negative	-	-
GPA	8	Negative	Negative	Negative	40.0	-

AAV: ANCA-associated vasculitis; BVAS: Birmingham Vasculitis Activity Score; EGPA: eosinophilic granulomatosis with polyangiitis; GPA: granulomatosis with polyangiitis; C-ANCA: cytoplasmic ANCA; PR3-ANCA: anti-proteinase 3 ANCA; IIF: indirect immunofluorescence; *iv*: intravenous.

Comparisons between AAV patients with active disease presenting or not PR3-ANCA yielded no significant results regarding the BVAS score [5.5 (2.75-8.25) *vs* 6.0 (3.25-11.75); P=0.885], daily prednisone dose [45.0 mg (9.37-60.0) *vs* 20.0 mg (2.5-40.0); P=0.471], or the use of cyclophosphamide/rituximab or no immunosuppressive/biologic agent (P=1.000). In five AAV patients treated with rituximab, IIF-ANCA positivity at diagnosis (two out of five AAV patients) remained the same using the standard technique and the IIF-ANCA improved technique.

## Discussion

This study was motivated by the overwhelmingly lower positivity rate of IIF-ANCA tests performed at the Immuno-Rheumatology Laboratory at a reference university hospital compared to IIF-ANCA and PR3- and MPO-ANCA tests performed in a CAP-certified private laboratory in the investigation of AAV and other diseases associated with ANCA. Our results pointed out that modifications in reagents used for the IIF-ANCA tests and maintenance of the microscope led to a significant improvement in the positivity rate and enhancement of IIF-ANCA titer in samples from patients with diseases associated with ANCA. However, neither the standard nor the improved IIF-ANCA tests displayed an acceptable agreement rate with MPO- and PR3-ANCA using third-generation ELISA. In addition, disease activity and therapy had no impact on results of ANCA tests in AAV patients.

There was a significant difference among the 3 groups included in this study regarding gender proportion, age, and time since diagnosis. These differences may reflect epidemiologic differences among AAV, type 1 AIH, and UC. Age and gender do not interfere in ANCA detection; however, time since diagnosis, might indirectly indicate a well-controlled and well-treated disease, which might lead to a lower ANCA detection (6,18,19). Nevertheless, when ANCA test results were evaluated at diagnosis, the frequently negative results observed in the Immuno-Rheumatology laboratory were not consistent with the expected positivity rate reported in literature, which reports sensitivity and specificity in AAV of 67-89 and 93%, respectively (5,20). That inconsistency had already been noted by the physicians following patients with AAV, and frequently additional ANCA tests were performed in other laboratories in order to obtain support for an AAV diagnosis. The variability of IIF-ANCA results among different laboratories is a well-known feature of this test, which may be due to the lack of standardization of the technique and to the successive operator-dependent steps of this test (21,22).

A study performed by the EUVAS (European Vasculitis Study Group) in 2016 compared IIF-ANCA techniques performed at two sites to eight solid-phase immunoassays for MPO- and PR3-ANCA. In the analysis of the receiver operating characteristic (ROC) curve, all solid-phase immunoassays had area under the curve similar to or greater (0.919-0.959) than those obtained for IIF-ANCA performed in the two laboratories (0.842-0.923). The comparison of IIF-ANCA tests performed at the two laboratories yielded a large variability in results for the same samples. It is worth noting that one laboratory used only ethanol-fixed slides while the other laboratory used both ethanol- and formalin-fixed slides for the IIF-ANCA. The use of both fixation methods had better sensitivity and specificity than the use of only ethanol-fixed slides, with an accuracy of 0.927 *vs* 0.794 (23). In another study performed by the same group, the manual technique was compared to automated IIF-ANCA. The manual IIF-ANCA technique had 89% sensitivity and 94% specificity in one laboratory, whereas sensitivity and specificity were 84 and 79%, respectively, in another laboratory. Thus, these findings stress the large inter-laboratory variability of IIF-ANCA results. Nonetheless, the use of automated IIF-ANCA assays still led to a high variability in results between different laboratories; this variability may be due to different substrates and algorithms used to define fluorescence patterns used by each commercially available automated IIF-ANCA (20).

In this study, simply changing the materials used to perform the IIF-ANCA technique and general maintenance of the microscope led to a significant increment in the positivity rate of IIF-ANCA results. The quality and reliability of in-house ethanol-fixed slides are highly influenced by the type of substrate and conjugated antibodies and by the fixation method used. In addition, slide incubation and washing steps, as well as the maintenance status of the fluorescence microscope, all seem to influence the IIF-ANCA test results. Thereby, interventions in materials and in the operational steps of IIF-ANCA technique may possibly explain the better performance of the improved IIF-ANCA technique (12,24). It is worth mentioning that ethanol-fixed slides displayed too many lymphocytes and few neutrophils in the standard IIF-ANCA, and that might be the result of the reagent used for polymorphonuclear granulocytes isolation. Hystopaque^®^ (Merck), used in the standard procedure, is specific for mononuclear cells, while PolymorphPrep™ (Progen), used after improvements in the IIF-ANCA technique, is specific for polymorphonuclear granulocytes (12).

Additionally, excessive background staining was observed in the slides in the standard IIF-ANCA technique, whereas in the improved technique the slides had few or no background staining. This difference may be due to the conjugated antibody used. Moreover, the fluorescence microscope itself may also interfere in IIF-ANCA results. In this study, the replacement of the filter, lamps, and objectives led to a better performance of the IIF-ANCA compared to the standard technique (12).

In the present study, slides with formalin-fixed neutrophils were also used to assess samples that tested positive for both P-ANCA and ANA. Although the 1999 International Consensus on ANCA Testing recommended only the use of slides with ethanol-fixed neutrophils (25), some argue that there is still a place for IIF-ANCA with formalin-fixed neutrophils to distinguish false positive P-ANCA from ANA positivity. Unfortunately, this technique is also associated with a high rate of false-positive results, since it is common to observe background fluorescence in neutrophils, even in negative samples (12). Conversely, other groups argue that IIF-ANCA with formalin-fixed neutrophils is an accurate technique with high sensitivity and specificity (18,24,26). In this study, only four samples had to be tested in slides with formalin-fixed neutrophils, all of which were only ANA positive.

We could not reach an acceptable agreement between IIF-ANCA and ELISA results. This finding could possibly be due to the remission state of most patients and the use of immunosuppressants. Differently from our approach, studies demonstrating high sensitivity of second and third generation anti-PR3/MPO immunoassays for the diagnosis of AAV included only patients with active disease at disease onset (27). Moreover, it is well-established that C-ANCA positivity lasts for a longer period of time than PR3-ANCA, and both MPO- and PR3-ANCA are usually present at higher titter at diagnosis and reduce after immunosuppressive therapy (19,27). Therefore, this dissociation between IIF-ANCA and ELISA results found in our study may be explained in part by the positive IIF-ANCA results lasting longer compared to the more ephemeral ELISA positivity.

The major limitation of our study was the lack of patients at disease onset, as all included patients had a long-term disease duration at study entry. Therefore, all samples assessed were collected from patients already under treatment and most of them in remission, which might have interfered in ANCA positivity. Although the association between ANCA positivity and disease activity in AAV is still controversial, positive ANCA tests are found more frequently and at higher titer in patients with active disease at diagnosis. Hence, there is some association between ANCA positivity, disease activity, and severity. Frequently, the ANCA test becomes negative when remission is attained and it often reappears before disease relapses or, for those that remained positive during the remission period, an increase in ANCA titer is detected prior to relapses (6,18,22,25,28). A study performed in Japan with MPO-ANCA positive patients found a negative conversion rate of 72% at remission within 6 months of induction therapy (29). In the RAVE study, 50% of PR3-ANCA positive patients treated with rituximab and 24% treated with cyclophosphamide tested negative during remission. In contrast, such difference was not detected on MPO-ANCA-positive patients, where the negative conversion rate was similar between rituximab and cyclophosphamide treatment groups (40 and 41%, respectively) (30).

Another potential limitation of our study is the relatively small number of patients with UC and type I AIH, which could reduce the real representativeness of both diseases regarding ANCA profile. Positivity rates of ANCA in these diseases, usually A-ANCA, reported for UC and type I AIH are 50-70% and 50-96%, respectively (6,31). On the other hand, the positivity rate of IIF-ANCA tests in our study increased from the standard to the improved technique from 5.3 to 36.8% for UC patients and from zero to 25% for type I AIH. Therefore, even after the improvements in IIF-ANCA, the positivity rate remained lower than that reported by the literature.

In conclusion, improvements applied to the IIF-ANCA technique led to a significant improvement in test sensitivity for patients with diseases associated to ANCA, as well as to a significant increment in median IIF-ANCA titer. Nonetheless, the agreement between IIF-ANCA and MPO- or PR3-ANCA was low with both the standard and improved techniques.

## Supplementary Material

Click to view [pdf].
